# Ergometrine stimulates histamine H_2_ receptors in the isolated human atrium

**DOI:** 10.1007/s00210-023-02573-8

**Published:** 2023-06-24

**Authors:** Hannes Jacob, Pauline Braekow, Britt Hofmann, Uwe Kirchhefer, Lisa Forster, Denise Mönnich, Laura J. Humphrys, Steffen Pockes, Joachim Neumann, Ulrich Gergs

**Affiliations:** 1 Institute for Pharmacology and Toxicology, Medical Faculty, Martin Luther University Halle-Wittenberg, Magdeburger Straße 4, 06097 Halle (Saale), Germany; 2 Department of Cardiac Surgery, Mid-German Heart Center, University Hospital Halle, Ernst Grube Straße 40, 06097 Halle (Saale), Germany; 3 Institute for Pharmacology and Toxicology, Medical Faculty, Westfälische Wilhelms- Universität Münster, Domagkstraße 12, 48149 Münster, Germany; 4 Institute of Pharmacy, University of Regensburg, Universitätsstraße 31, 93040 Regensburg, Germany

**Keywords:** Ergometrine, Human atrium, Mouse atrium, Mouse ventricle

## Abstract

Ergometrine (6a*R*,9*R*)-*N*-((*S*)-1-hydroxypropan-2-yl)-7-methyl-4,6,6a,7,8,9-hexa-hydro-indolo-[4,3-*fg*]chinolin-9-carboxamide or lysergide acid β-ethanolamide or ergonovine) activates several types of serotonin and histamine receptors in the animal heart. We thus examined whether ergometrine can activate human serotonin 5-HT_4_ receptors (h5-HT_4_R) and/or human histamine H_2_ receptors (hH_2_R) in the heart of transgenic mice and/or in the human isolated atrium. Force of contraction or beating rates were studied in electrically stimulated left atrial or spontaneously beating right atrial preparations or spontaneously beating isolated retrogradely perfused hearts (Langendorff setup) of mice with cardiac specific overexpression of the h5-HT_4_R (5-HT_4_-TG) or of mice with cardiac specific overexpression of the hH_2_R (H_2_-TG) or in electrically stimulated human right atrial preparations obtained during cardiac surgery. Western blots to assess phospholamban (PLB) phosphorylation on serine 16 were performed. Ergometrine exerted concentration- and time-dependent positive inotropic effects and positive chronotropic effects in atrial preparations starting at 0.3 µM and reaching a plateau at 10 µM in H_2_-TGs (*n* = 7). This was accompanied by an increase in PLB phosphorylation at serine 16. Ergometrine up 10 µM failed to increase force of contraction in left atrial preparations from 5-HT_4_-TGs (*n* = 5). Ten micrometer ergometrine increased the force of contraction in isolated retrogradely perfused spontaneously beating heart preparations (Langendorff setup) from H_2_-TG but not 5-HT_4_-TG. In the presence of the phosphodiesterase inhibitor cilostamide (1 µM), ergometrine at 10 µM exerted positive inotropic effects in isolated electrically stimulated human right atrial preparations, obtained during cardiac surgery, and these effects were eliminated by 10 µM of the H_2_R antagonist cimetidine but not by 10 µM of the 5-HT_4_R antagonist tropisetron. Furthermore, ergometrine showed binding to human histamine H_2_ receptors (at 100 µM and 1 mM) using HEK cells in a recombinant expression system (p*K*_i_ < 4.5, *n* = 3). In conclusion, we suggest that ergometrine is an agonist at cardiac human H_2_Rs.

## Introduction

**Fig. 1:**
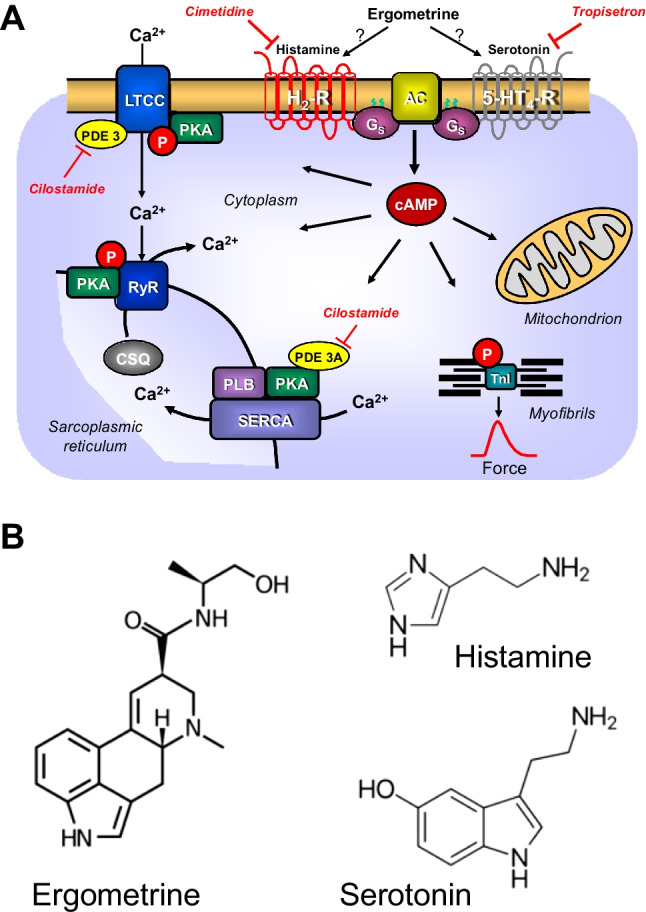
**(A)** Scheme of possible mechanisms of action of ergometrine in cardiac myocytes. 5-HT_4_-R, type 4 serotonin receptor (can be blocked by tropisetron); cAMP, 3,5-cyclic adenosine monophosphate; CSQ, calsequestrin; Gs, stimulatory G-protein; H_2_-R, type 2 histamine receptor (can be blocked by cimetidine); LTCC, L-type Ca^2+^ channel; P, phosphorylation; PDE3, phosphodiesterase 3 (the activity can be blocked by cilostamide); PKA, cAMP-dependent protein kinase; PLB, phospholamban; TnI, inhibitory subunit of troponin; RYR, ryanodine receptors; SERCA, sarcoplasmic reticulum Ca^2+^-ATPase. **(B)** Structural formulae of ergometrine, histamine and serotonin

There are four histamine receptors in the mammalian heart at an RNA and/or protein level (review: Neumann et al. 2021a). In the hearts of the wild type mouse, rat, dog and cat, the positive inotropic effect of histamine has been found to be indirect via release of endogenous catecholamines (Flacke et al. 1967; Dai et al. 1976; Bartlet 1963; Wellner-Kienitz et al. 2003, Gergs et al. 2019, 2020, 2021b, Neumann et al. 2021b, 2021c, 2021e, Laher und McNeill 1980a, 1980b). In human hearts, histamine H_2_ receptors (H_2_R) are expressed in the atrium and ventricle (Fig. 1A, radioligand binding: Baumann et al. 1982, 1983, 1984, antibody and RNA expression: Matsuda et al. 2004). In isolated human atrial cardiac preparations, H_2_Rs mediate a positive inotropic effect of exogenously applied histamine (Levi et al. 1981, Genovese et al. 1988, Zerkowski et al. 1993, Sanders et al 1996, Thoren et al. 2011). To create a model of H_2_Rs in the human heart, we have produced transgenic mice that overexpress the human H_2_R in the heart (H_2_-TG). In these transgenic mice, histamine can increase the force of contraction in atrial and ventricular preparations (Gergs et al. 2019, 2020, 2021a, Neumann et al. 2021b, c, d, e).

Ergometrine (Fig. 1B) is found in fungi-like *Secale cornutum* and is mainly used in the clinic for the treatment of bleeding after childbirth. It is important to understand the pharmacology of ergometrine better as it stands on the list of essential drugs of the World Health Organization (WHO 2019). Ergometrine can activate serotonin 5-HT_2A_ receptors (5-HT_2A_R) in the brain, which may lead to its hallucinogenic effect (Cortijo et al. 1997). Ergometrine can also lead to vasoconstriction, possibly due to ergometrine stimulating peripheral vascular 5-HT_2A_R and α_1_-adrenoceptors (review: Silberstein and McCrory 2003). It has also been shown that ergometrine increased the force of contraction in the guinea pig heart. Guinea pig ventricles contain functional H_2_R (Bongrani et al. 1979, review: Neumann et al. 2021a). As far as we know, a positive inotropic cardiac effect by ergometrine in human cardiac preparations has never been published before.

As ergometrine contains a tryptamine ring in its chemical structure and binds to the 5-HT_2A_R (Cortijo et al. 1997), it was conceivable that ergometrine activated human serotonin receptors in the heart. In the human heart, all inotropic and chronotropic effects of serotonin are not mediated via 5-HT_2A_Rs but through 5-HT_4_Rs (reviews: Kaumann and Levy 2006; Neumann et al. 2017, 2023). These 5-HT_4_R are lacking in function in the mouse heart (as described above for histamine): i.e. serotonin does not increase force of contraction in isolated mouse cardiac preparations from wild type mice (WT, Gergs et al. 2010, 2013; Neumann et al. 2019, 2021b, 2021e). To facilitate the study of human 5-HT_4_Rs, we produced and characterized a transgenic mouse with cardiac specific overexpression of this receptor (5-HT_4_-TG), which responds with positive inotropic and chronotropic effects to exogenously applied serotonin (Gergs et al. 2010; review: Neumann et al. 2017, 2023).

Hence, we tested the following hypotheses: ergometrine may increase force of contraction in cardiac preparations of 5-HT_4_-TG and/or H_2_-TG and in human atrial preparations. Preliminary results have been presented in abstract form (Jacob et al. 2023).

## Materials and methods

### Transgenic mice

The investigation conforms to the Guide for the Care and Use of Laboratory Animals published by the National Research Council (2011). Animals were maintained and handled according to approved protocols of the animal welfare committees of the University of Halle-Wittenberg, Germany. The generation and initial characterization of the transgenic mice were described before (Gergs et al. 2010, 2019). In brief, the human H_2_R cDNA or the human 5-HT_4_R cDNA was inserted into a mouse cardiac α-myosin heavy chain promoter expression cassette. For all experiments, adult transgenic mice and WT littermates of both sexes were used.

### Contractile studies in mice

As described before, the right or left atrial preparations from the mice were isolated and mounted in organ baths (Gergs et al. 2013; Neumann et al. 1998). The bathing solution of the organ baths contained 119.8 mM NaCI, 5.4 mM KCI, 1.8 mM CaCl_2_, 1.05 mM MgCl_2_, 0.42 mM NaH_2_PO_4_, 22.6 mM NaHCO_3_, 0.05 mM Na_2_EDTA, 0.28 mM ascorbic acid and 5.05 mM glucose. The solution was continuously gassed with 95% O_2_ and 5% CO_2_ and maintained at 37 °C and pH 7.4 (Neumann et al. 1998, Kirchhefer et al. 2004). Spontaneously beating right atrial preparations from mice were used to study any chronotropic effects. The drug application was as follows. After equilibration, ergometrine was cumulatively added to left atrial or right atrial preparations to establish concentration–response curves. Then, where indicated, either serotonin or histamine was additionally applied to the preparations. In separate experiments, concentration–response curves for ergometrine in mouse left atrial preparations were obtained, and after the effect of 10 µM ergometrine had reached a plateau the atrial strips were flash frozen with liquid nitrogen for further study.

### Contractile studies on human preparations

The contractile studies on human preparations used the same setup and buffer as used in the mouse studies. The samples were obtained from 3 male patients and 4 female patients, 78–82 years. Drug therapy included atorvastatin, spironolactone, amlodipine, ticagrelor, lisinopril, pantoprazole, sacubitril/valsartan, metoprolol, furosemide, torasemide, apixaban and acetyl salicylic acid. Patients had been diagnosed with NYHA II-III and CCS I-III. Our methods used for atrial contraction studies in human samples have been previously published and were not altered in this study (Gergs et al. 2009, 2021b). Written informed consent was obtained for the use of right atrial tissues from patients undergoing cardiac surgery.

### Isolated perfused hearts

As described by our group (Dörner et al. 2021; Gergs et al. 2004, 2010, 2019), isolated whole mouse hearts were retrogradely perfused with the same buffer as in Sect. 2.2. above. Hearts were allowed to beat by themselves. Force was monitored from the apex cordis by a hook connected to an electronic force monitor and digitized. Perfusion with drugs took place with a syringe connected to a pump. This pump was connected as a bypass with the aorta. At the end of experiments, hearts were freeze-clamped in liquid nitrogen to stop any phosphorylation reactions. Frozen samples were kept at − 80 °C until biochemical analysis.

### Western blotting

The homogenization of the samples, protein measurements, electrophoresis, primary and secondary antibody incubation and quantification were performed following our previously established protocols (Gergs et al. 2009, 2019; 2020). Primary antibodies were anti-calsequestrin (CSQ) antibody, #ab3516, abcam, Cambridge, UK (diluted 1:20,000) and anti-phospholamban (pSer16) antibody, #A-010–12, Badrilla, Leeds, UK (diluted 1:5000).

### Radioligand competition binding

Radioligand competition binding experiments were performed as previously described by using the HEK293-SP-FLAG-hH_2_R cell line and [^3^H]UR-DE257 (*K*_d_ = 66.9 nM, c = 40 nM) (Pockes et al. 2018; Rosier et al. 2021; Baumeister et al. 2015). Ligand dilutions were prepared tenfold concentrated in L-15 with 1% BSA, and 10 μL/well was transferred to a flat-bottom polypropylene 96-well microtiter plate (Greiner Bio-One, Frickenhausen, Germany), as well as 10 μL/well of the respective radioligand. The cells were adjusted to a density of 1.25 × 10^6^ cells/mL, and 80 μL of the cell suspension was added to each well (total volume of 100 μL). All data were analyzed using GraphPad Prism 9 software (San Diego, CA, USA). The normalized competition binding curves were then fitted with a four-parameter logistic fit yielding pIC_50_ values. These were transformed into p*K*_i_ values using the Cheng − Prusoff equation (Cheng and Prusoff 1973).

### Data analysis

Data shown are means ± standard error of the mean. Statistical significance was estimated using Student’s *t* test or, for multiple comparisons, the analysis of variance followed by Bonferroni’s *t* test, as appropriate. A *p* value < 0.05 was considered to be significant.

### Drugs and materials

Ergometrine was in dissolved dimethylsulfoxide (DMSO); serotonin and histamine were dissolved in water and were purchased from Sigma-Aldrich (Germany). All other chemicals were of the highest purity grade commercially available. Deionized water was used throughout the experiments. Stock solutions were prepared fresh daily.

## Results

### H_2_-TG left atrial preparations

**Fig. 2:**
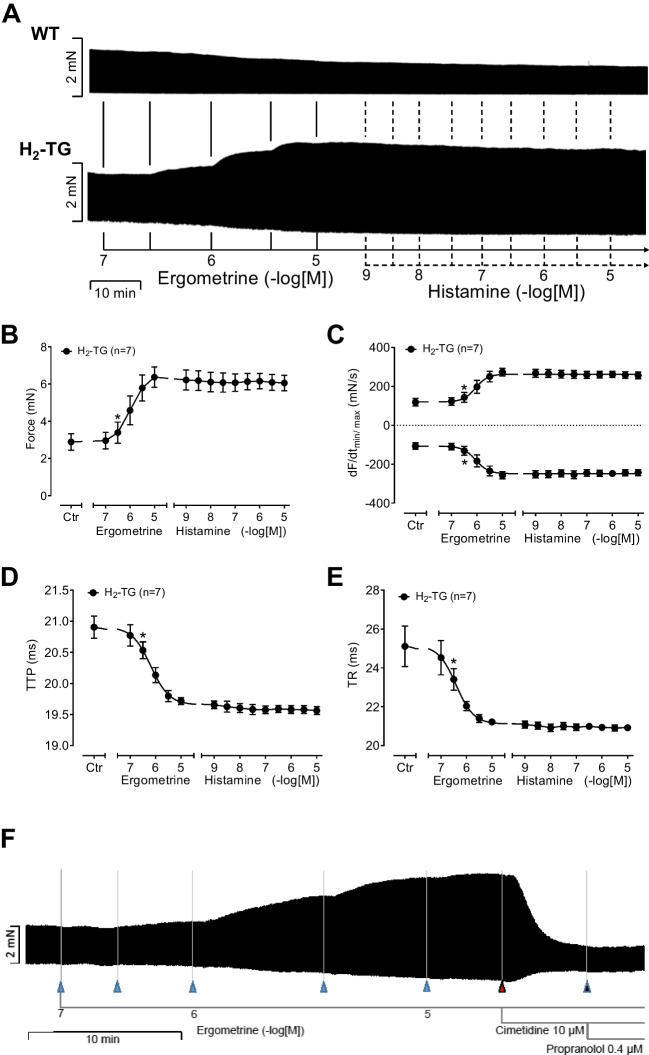
**(A)** Original recordings of mouse left atrial preparations from WT and H_2_-TG. Ergometrine induced a positive inotropic effect only in H_2_-TG, but not in WT. **(B–E)** Summarized concentration–response curves for the effect of ergometrine and additionally applied histamine on force of contraction **(B)**, maximum rate of tension development (dF/dt_max_) and rate of tension relaxation (dF/dt_min_) **(C)**, time to peak tension (TTP) **(D)** and time to relaxation (TR) **(E)**. (**F**) Original recording of a murine H_2_-transgenic left atrial preparation showing a concentration–response curve of ergometrine, followed by the application of 10 µM cimetidine and 0.4 µM propranolol. Positive inotropic effect of ergometrine was reversed by cimetidine but not propranolol. *p < 0.05 vs. Ctr, first significant difference versus Control (pre-drug value, Ctr). “*n*” indicates number of experiments

We noticed that ergometrine time and concentration dependently increased the force of contraction in H_2_-TG. A typical original recording is included as Fig. 2A. For comparison, we studied WT. In WT, ergometrine failed to increase the force of contraction (Fig. 2A). In H_2_-TG, additionally applied histamine failed to increase force of contraction further (Fig. 2A), while in the left atrium additionally applied histamine was ineffective to augment the force of contraction from WT (Fig. 2A). On the other hand, ergometrine concentration dependently increased force of contraction in left atrial preparations (Fig. 2B). Moreover, ergometrine concentration dependently shortened the time to peak tension (Fig. 2D). This shortening was so extensive that additionally applied histamine could not shorten relaxation any further. In a similar fashion, ergometrine hastened time of relaxation concentration dependently, and additionally applied histamine was not more effective than ergometrine (Fig. 2E). In addition, ergometrine also enhanced the absolute value of the rate of tension development and the absolute rate of tension relaxation (Fig. 2C). Again, additionally applied histamine failed to augment the absolute values of the rates of tension development any further (Fig. 2C).

We also investigated the effect of the antagonists at potentially involved receptors, i.e. cimetidine and propranolol for H_2_R and β-adrenoceptor. The positive inotropic effect of previously applied 10 µM of ergometrine was shown to be reversible by additionally applied 10 µM of cimetidine, whereas additionally applied propranolol showed no further effect, as can be seen in Fig. 2F.

### H_2_-TG right atrial preparations

**Fig. 3:**
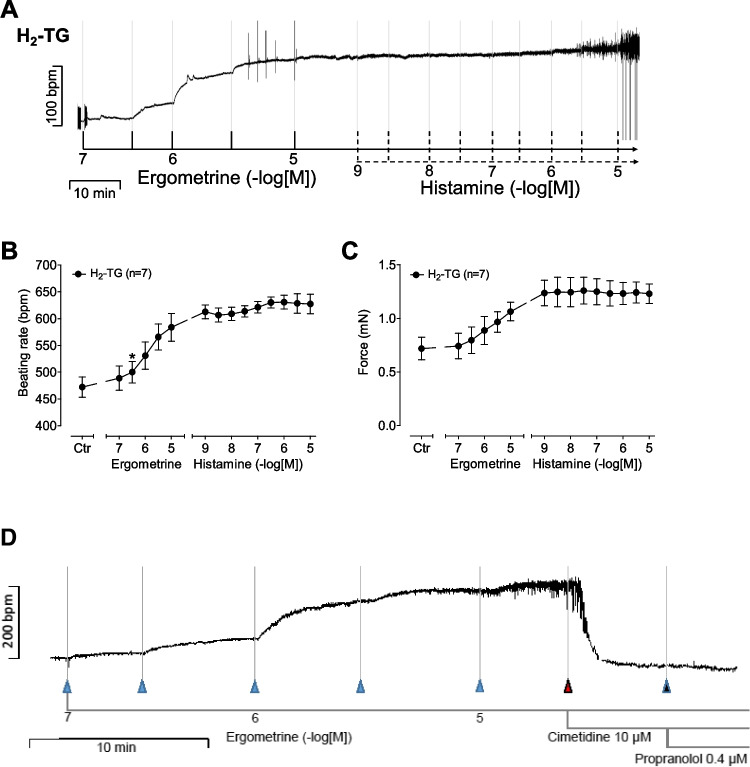
**(A)** Original recording: Effect of ergometrine and additionally applied histamine on beating rate in spontaneously beating right atrial preparations from H_2_-TG. **(B)** Summarized effect of ergometrine and additionally added histamine on beating rate in beats pro minute (bpm) and **(C)** on force of contraction in milli Newton (mN) in spontaneously beating right atrial preparations from H_2_-TG. (**D**) Original recording of a murine H_2_-transgenic right atrial preparation showing a concentration–response curve of ergometrine, followed by the application of 10 µM cimetidine and 0.4 µM propranolol. Positive chronotropic effect of ergometrine was reversed by cimetidine but not propranolol. *p < 0.05, First significant differences versus control (Ctr; pre-drug value). “*n*” indicates number of experiments

In right atrial preparations from H_2_-TG, ergometrine increased the beating rate time and concentration dependently, as seen in an original recording (Fig. 3A). Additionally applied histamine did not increase the beating rate any further (Fig. 3A). Several such experiments are summarized in Fig. 3B for the effect of ergometrine on beating rate or force of contraction (Fig. 3C). Additionally applied histamine did not augment the beating rate or force of contraction further (Fig. 3A, B, C).

We also investigated the effect of the antagonists at potentially involved receptors, i.e. cimetidine and propranolol for H_2_R and β-receptors. The positive chronotropic effect of previously applied 10 µM of ergometrine was shown to be reversible by additionally applied 10 µM of cimetidine, whereas additionally applied propranolol showed no further effect, as can be seen in Fig. 3D.

### 5-HT_4_-TG left atrial preparations

**Fig. 4:**
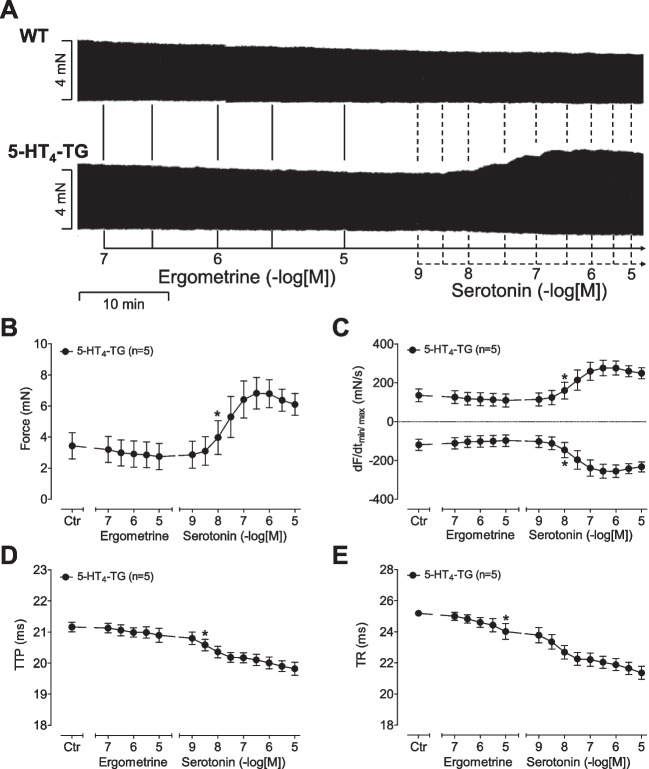
**(A)** Original recordings of mouse left atrial preparations from WT and 5-HT_4_-TG. It becomes apparent that ergometrine is unable to induce an inotropic effect neither in WT nor in 5-HT_4_-TG left atrium, whereas serotonin induced a positive inotropic effect only in 5-HT_4_-TG. **(B–E)** Summarized concentration–response curves for the effect of ergometrine or additionally applied serotonin on force of contraction in milli Newton (mN) **(B)**, maximum rate of tension development (dF/dt_max_) and rate of tension relaxation (dF/dt_min_) in milli Newton per second (mN/s) **(C)**, time to peak tension (TTP) **(D)** and time to relaxation (TR) in milli seconds (ms) **(E)**. *p < 0.05 vs. Ctr, first significant difference versus Control (pre-drug value, Ctr). “*n*” indicates number of experiments

We noticed that ergometrine did not increase the force of contraction in atrial preparations from 5-HT_4_-TG or WT. Typical original recordings are presented in Fig. 4A. In 5-HT_4_-TG, additionally applied serotonin (5-HT) increased force of contraction (Fig. 4A), whereas in WT 5-HT failed to augment force of contraction (Fig. 4A). Summarizing the results, ergometrine did not increase force of contraction in left atrial preparations from 5-HT_4_-TG (Fig. 4B). Moreover, ergometrine failed to shorten the time to peak tension in 5-HT_4_-TG (Fig. 4D). Additionally applied serotonin, in contrast, shortened the time to peak tension. In a similar fashion, ergometrine did not significantly affect the time of relaxation but additionally applied serotonin was effective to shorten the time of relaxation in 5-HT_4_-TG (Fig. 4E). In addition, ergometrine did not enhance the absolute values of the rates of tension development and relaxation in 5-HT_4_-TG (Fig. 4C). However, subsequently applied serotonin increased both the rate of tension development and relaxation (Fig. 4C).

### 5-HT_4_-TG right atrial preparations

**Fig. 5:**
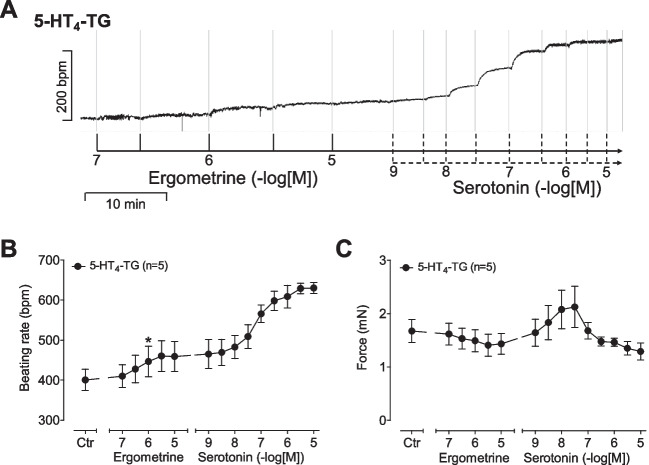
**(A)** Original recording: Effect of ergometrine and additionally applied serotonin on beating rate in spontaneously beating right atrial preparations from 5-HT_4_-TG. **(B)** Summarized effect of ergometrine and additionally added serotonin on beating rate in beats pro minute (bpm) and **(C)** on force of contraction in milli Newton (mN) in spontaneously beating right atrial preparations from 5-HT_4_-TG. *p < 0.05, First significant differences versus control (Ctr; pre-drug value). “*n*” indicates number of experiments

In right atrial preparations from 5-HT_4_-TG, ergometrine hardly increased the beating rate as seen in an original recording (Fig. 5A). However, looking closely at the data, there is a tendency of ergometrine to increase beating rate in the original recording (Fig. 5A), and in the summarized data, this increase was a little bit clearer (Fig. 5B). We hypothesized that the effect of ergometrine on mouse right atrial preparations from 5-HT_4_-TG might be due to stimulation of murine H2-R in the sinus node. Additionally applied serotonin increased the beating rate in 5-HT_4_-TG (Fig. 5A). Several such experiments are summarized in Fig. 5B. In the same right atrial preparations, we also quantified the mechanical parameters: Here, similar to the findings in left atrial preparations, ergometrine did not increase force of contraction (Fig. 5C), whereas additionally applied serotonin did. It may be asked why higher concentrations of 5-HT reduced the force of contraction (Fig. 5C). These are likely indirect effects: higher concentrations of 5-HT concentration dependently increased the beating rate (Fig. 5B). Mice exhibit a negative staircase or “Treppe” phenomenon. In other words, an increase in the beating rate by itself reduces the force of contraction in the mouse atrium. Hence, it is plausible that these frequency-dependent negative inotropic effects of 5-HT overcome any direct positive inotropic effects of 5-HT via 5-HT_4_Rs in right atrial preparations.

### WT right atrial preparations

**Fig. 6:**
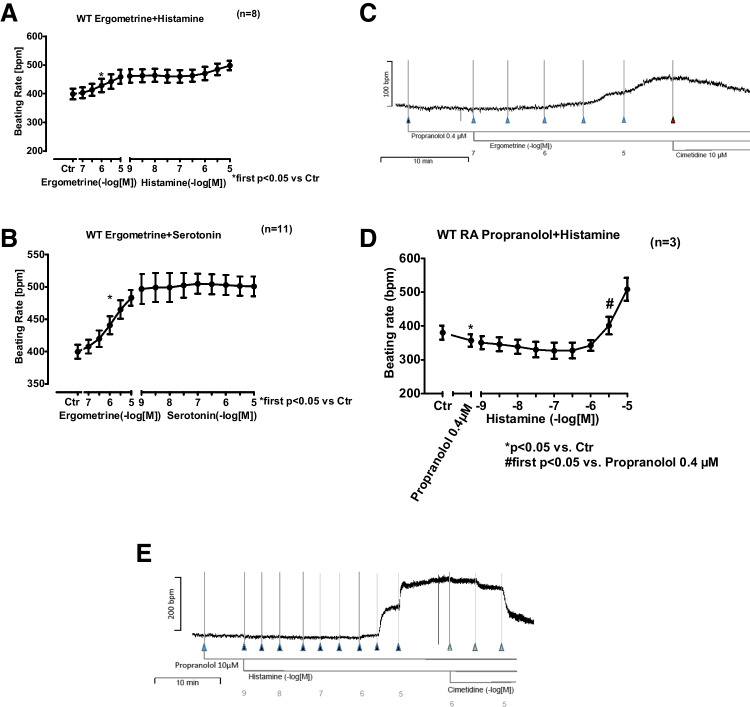
**(A)** Summarized effect of ergometrine and additionally applied histamine on murine WT right atrial preparations: Ergometrine and histamine significantly increase the beating rate. **(B)** Summarized effect of ergometrine and additionally applied serotonin on murine WT right atrial preparations: Ergometrine but not serotonin increased the beating rate. **(C)** Original recording of a concentration–response curve of ergometrine in the presence of 0.4 µM of propranolol: positive chronotropic effect reversible by additionally applied 10 µM of cimetidine. **(D)** Summarized effect of histamine in the presence of 0.4 µM propranolol on WT murine right atrial preparations: Histamine significantly increased the beating rate. **(E)** Original recording of a concentration–response curve of histamine in the presence of propranolol on a WT murine right atrial preparation: Histamine increased the beating rate; this was reversible by additionally applied 10 µM of cimetidine. *p < 0.05, First significant differences versus control (Ctr; pre-drug value). “*n*” indicates number of experiments

Ergometrine (100 nM–10 µM) caused an increase in the beating rate of both WT and 5-HT_4_-transgenic murine right atrial preparations, where the human H_2_-receptor is absent. We hypothesized that this positive chronotropic effect of ergometrine on WT and 5-HT4-TG murine right atrial preparations might be mediated by murine H2-receptors in the sinus node. Ergometrine and additionally applied histamine (1 nM–10 µM) increased the beating rate of right atrial preparations from WT (Fig. 6A), while additionally applied serotonin (1 nM–10 µM) did not increase the beating rate further (Fig. 6B). We investigated the effect of antagonists at potentially involved receptors, i.e. cimetidine and propranolol for H_2_ and β-receptors, as can be seen in Fig. 6C. The beating rate of murine WT right atrial preparations was increased by 10 µM of ergometrine despite the presence of previously applied 0.4 µM of propranolol. This positive inotropic effect could be reversed by additionally applied 10 µM of cimetidine, suggesting the involvement of enogenous mouse H_2_ R- but not β-receptors.

In order to further test that hypothesis we conducted experiments with histamine (1 nM–10 µM) in the presence of 10 µM of propranolol on right atrial preparations from WT and found that histamine at concentrations of 3 µM and above caused a positive chronotropic effect of 43% (± 10.9%) at 10 µM relative to Ctr (*p* < 0.05). These data can be found in Fig. 6D. Moreover, this positive chronotropic effect of histamine could be reversed by 10 µM of cimetidine, as can be seen in Fig. 6E. Thus, we conclude that the effect of high concentrations of histamine on the beating rate of WT right atrial preparations might be due to H2-R rather than β-adrenoceptors.

### Isolated perfused hearts

It was interesting to study whether ergometrine affects ventricular function because the human ventricle rather than the atrium is mainly responsible for the cardiac output. Isolated human ventricular preparations were not available to us. Instead, to get an insight into ventricular actions of ergometrine, we used the Langendorff preparation, a spontaneously beating retrogradely buffer-perfused heart from the mouse. Here, force of contraction from the apex cordis was quantified under isometric conditions. We perfused hearts of H_2_-TG, 5-HT_4_-TG and WT through the coronary arteries with ergometrine (10 µM). In brief, 10 µM ergometrine increased force of contraction and relaxation rate in hearts from H_2_-TG, but not from 5-HT_4_-TG and WT (Table 1).

**Table 1:** Effect of ergometrine (10 µM) on force of contraction in milli Newton (mN) and on the time to relaxation (TR) in milliseconds (ms) in isolated spontaneously beating retrogradely buffer perfused hearts (Langendorff mode) from H_2_-TG, 5-HT_4_-TG and WT. *N* gives the number of experiments

	WT	H_2_-TG	5-HT_4_-TG
*N* =	5	5	5
Basal force (mN)	12.9 ± 1.4	8.5 ± 1.7	10.1 ± 2.9
After ergometrine (mN)	13.2 ± 1.4	15.5 ± 2.6 ^#^	11.6 ± 3.2
TR basal (ms)	68.2 ± 5.4	58.2 ± 3.5	65.3 ± 5.1
TR after ergometrine (ms)	67.9 ± 6.3	48.1 ± 3.8 ^#^	61.5 ± 4.8

^#^*p* < 0.05 vs. basal

### Human atrial contraction

We also investigated the effect of ergometrine on human right atrial samples obtained from bypass surgery.

**Fig. 7:**
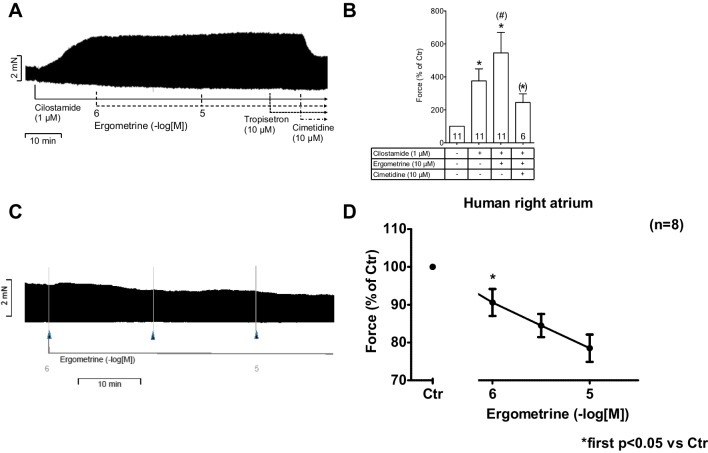
**(A)** Original recording of the effect of 1 and 10 µM ergometrine, 10 µM tropisetron and 10 µM cimetidine in the presence of 1 µM cilostamide on a human atrial preparation. **(B)** Summarized data of force of contraction in % of control (Ctr, pre-drug value). **(C)** Original recording of the effect of 1 to 10 µM ergometrine without preincubation with cilostamide on human right atrial tissue: no positive inotropic effect. **(D)** Summarized effect of 1 to 10 µM of ergometrine on human right atrial tissue without preincubation with cilostamide. Further data are listed in Table 2. *p < 0.05 vs. Ctr (one-way analysis of variance (ANOVA)), ^(^*^)^*p* < 0.05 vs. Ctr (*t* test), ^(#)^*p* < 0.05 vs. cilostamide (*t* test), numbers in bars indicate the number of experiments

Initially, we investigated the effect of ergometrine (1 µM–10 µM) without any preincubation with cilostamide and found no positive inotropic effect of ergometrine. An original recording of such an experiment can be found in Fig. 7C, while data are summarized in Fig. 7D.

Therefore, we decided to pre-stimulate the samples with 1 µM of the selective phosphodiesterase III-inhibitor cilostamide and measure whether additionally applied ergometrine might raise the force of contraction any further. Phosphodiesterase III is the main isoenzyme in the human heart, and therefore we inhibited it here.

In isolated electrically stimulated right atrial preparations, cilostamide, per se (compare Fig. 1A), raised force of contraction (Fig. 7A). After this pre-stimulation, additionally applied ergometrine increased the force of contraction further. This is exemplified in the original recording depicted in Fig. 7A, while summarized data can be found in Fig. 7B and in Table 2. Likewise, the time to peak tension and time of relaxation were shortened by cilostamide and further reduced by ergometrine (Table 2). Similarly, the absolute values of the rates of tension development and relaxation development were increased by cilostamide and further enhanced by ergometrine (Table 2). Thereafter, the question arose whether these effects were mediated via H_2_Rs or 5-HT_4_Rs. Therefore, we additionally applied the respective antagonists (Table 2): tropisetron to block 5-HT_4_Rs and cimetidine to block H_2_Rs. As depicted in Table 2, the positive inotropic effect of ergometrine in isolated electrically stimulated human right atrial preparations was not sensitive to tropisetron but sensitive to additionally applied cimetidine. Likewise, the effect of ergometrine on the time to peak tension, time of relaxation as well as on the absolute values of the rates of tension and relaxation development was shown to be reversed by cimetidine, but not tropisetron (Table 2). Hence, we would regard this positive inotropic effect of ergometrine in the human right atrium as H_2_R mediated.

**Table 2:** Effect of ergometrine (10 µM), tropisetron (10 µM) and cimetidine (10 µM) in the presence of cilostamide (1 µM) on a human atrial preparation (number of experiments = 11; 6 for cimetidine)

	Force (mN)	TTP (ms)	TR (ms)	dF/dt_max_ (mN/s)	dF/dt_min_ (mN/s)
Ctr	1.07 ± 0.25	55.01 ± 3.45	134.57 ± 5.64	20.18 ± 4.94	− 10.09 ± 2.43
Cilostamide (1 µM)	2.75 ± 0.42*	50.98 ± 2.34	107.77 ± 5.97*	52.06 ± 8.37*	− 31.48 ± 5.06*
+ Ergometrine (10 µM)	3.93 ± 0.70**	49.19 ± 1.79	97.64 ± 4.46**	76.75 ± 13.77**	− 47.14 ± 8.29**
+ Tropisetron (10 µM)	4.01 ± 1.49	50.14 ± 2.28	91.02 ± 1.55	76.12 ± 27.64	− 47.14 ± 15.42
+ Cimetidine (10 µM)	2.61 ± 0.7***	52.61 ± 1.9***	107.05 ± 7.23	48.62 ± 14.8***	− 30.09 ± 9.3***

*Ctr* control (pre drug value), *dF/dt*_*max*_ maximum rate of tension development, *dF/dt*_*min*_ maximum rate of tension relaxation, *TR* time to relaxation, *TTP* time to peak tension

^*^p < 0.05 vs. Ctr

^**^p < 0.05 vs. Cilostamide

^***^p < 0.05 vs. Ergometrine

### Phosphorylation in mouse atrium

**Fig. 8:**
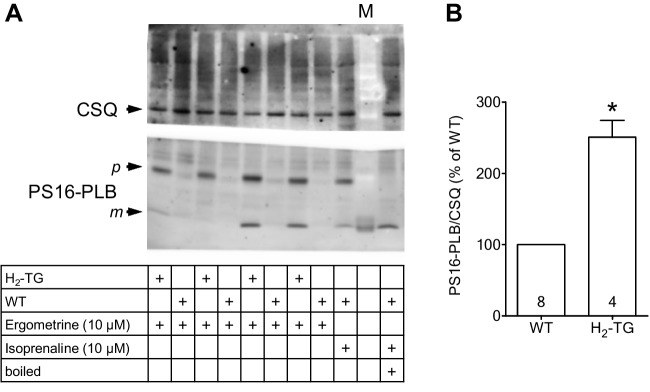
Ergometrine increased phosphorylation of phospholamban at serine-16 (PS16-PLB) in isolated mouse atrium from H_2_-TG but not from WT. **(A)** Typical Western blots are presented for PS16-PLB and cardiac calsequestrin (CSQ) as loading control. Contracting left atrial preparations from H_2_-TG or WT as seen in Fig. 2 were freeze-clamped and treated as described in Materials and Methods. Beta-adrenergic stimulation by isoprenaline was used as positive control, and one sample was boiled to show conversion of PLB from a higher (p, pentameric) to a lower (m, monomeric) molecular weight form. This mobility shift is typical for PLB. M, molecular weight marker. **(B)** Several experiments were quantified. In the ordinate, the ratio of the signal for PS16-PLB divided by CSQ was plotted after stimulation with ergometrine in H_2_-TG and WT (set as 100%) left atrial preparations. Numbers in bars indicate the number of experiments. *p < 0.05 vs. WT

In order to understand the signal transduction pathway of ergometrine better (compare Fig. 1A), we tested in separate experiments the effect of ergometrine on the phosphorylation state of phospholamban in heart samples from H_2_-TG and WT. Using western blot to analyze protein phosphorylation, 10 µM ergometrine increased the phosphorylation state of phospholamban in left atria of H_2_-TG but not WT (Fig. 8). An original image of a western blot can be seen in Fig. 8A, while the findings were summarized in Fig. 8B. Hence, ergometrine might stimulate signal transduction (Fig. 1A).

### Radioligand competition binding at the hH_2_R

**Fig. 9:**
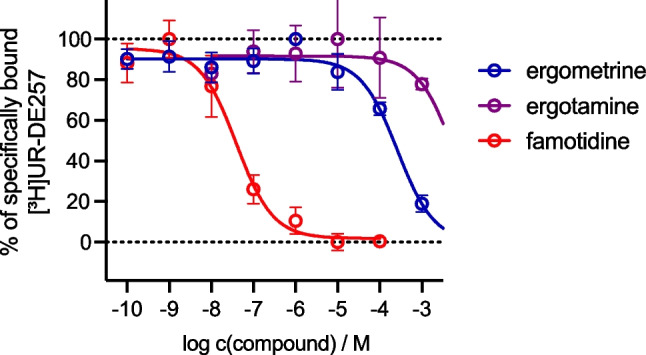
Displacement curves from representative radioligand competition binding experiments performed with ergometrine, ergotamine, famotidine (reference) and [^3^H]UR-DE257 (*K*_d_ = 66.9 nM, c = 40 nM) using HEK293-SP-FLAG-hH2R cells

We performed radioligand binding experiments at the human histamine H_2_ receptor (hH_2_R) using the HEK293-SP-FLAG-hH_2_R cell line and H_2_R radioligand [^3^H]UR-DE257 in competition with ergometrine, ergotamine and H_2_R reference antagonist famotidine. Ergometrine showed binding to H_2_R at 100 µM and 1 mM (p*K*_i_ < 4.5, *n* = 3), whereas for ergotamine almost no binding to the receptor could be measured up to a concentration of 1 mM (*n* = 3) (Fig. 9).

## Discussion

### Main new findings

The main new findings in this report consist firstly in the observation that ergometrine can act as a functional agonist on human H_2_Rs in the heart of an appropriate transgenic mouse model. Likewise, secondly we noted here that ergometrine only uses H_2_Rs to increase contractility in the isolated human atrium.

### 5-HT_4_-receptors

Looking at the chemical structure of ergometrine and knowing that ergometrine can act on 5-HT_2A_Rs in the periphery, we hypothesized that ergometrine might stimulate human cardiac serotonin receptors. In the human heart, serotonin only increases force via 5-HT_4_Rs. Hence, we thought ergometrine might stimulate 5-HT_4_ receptors in the human heart. As a first step we used our 5-HT_4_-TG mice as a model (Gergs et al. 2010). However, we did not note a significant positive inotropic effect of ergometrine in the atrium and ventricle of 5-HT_4_-TG animals. In atrial preparations, we could show that after ergometrine, serotonin could stimulate 5-HT_4_Rs because additionally applied serotonin raised the force of contraction further. The observations were similar in the right atrium; here, ergometrine was likewise inefficient, whereas serotonin increased force of contraction and the beating rate.

### H_2_-receptors

Considering the structural formula of ergometrine (Fig. 1B), an azole ring similar to the imidazole ring in histamine is identifiable. In addition, others noted that ergometrine can activate cardiac H_2_Rs in guinea pig Langendorff-perfused hearts (Bongrani et al. 1979). Hence, we thought it worthwhile to test ergometrine in our H_2_-TG model system (Gergs et al. 2019). We noted that in H_2_-TG, as in guinea pig, ergometrine acted as a functional agonist in concern to force of contraction and beating rate. Moreover, as additionally applied histamine in atrial preparations from H_2_-TG did not increase force or beating rate beyond the previous effect of ergometrine itself, we would thus suggest ergometrine should be regarded as a full functional agonist at human H_2_Rs expressed in the heart of H_2_-TG. Furthermore, ergometrine showed binding to human histamine H_2_ receptors (at 100 µM and 1 mM) using HEK cells in a recombinant expression system (p*K*_i_ < 4.5, *n* = 3, Fig. 8).

### Role of phosphorylation of regulatory proteins

The general assumption is that H_2_R stimulation leads to an increase in the phosphorylation state of proteins that are substrates for cAMP-dependent protein kinases (Fig. 1A, Neumann et al. 2021b). Indeed, we described that histamine acting via H_2_Rs can increase the phosphorylation state of phospholamban in the isolated human atrium and in the isolated atrium from H_2_-TG (Gergs et al. 2019; Neumann et al. 2021a). We extend here our previous studies with histamine by showing the ergometrine increases the phosphorylation state of phospholamban in H_2_-TG. This phosphorylation can explain, at least in part, why ergometrine increased the relaxation rate in atrial and ventricular preparations from H_2_-TG.

### Clinical relevance

Clinically, ergometrine (ergonovine) is sometimes used to detect Prinzmetal angina. In such patients, an increased heart rate was noted, which fits with our mouse data (H_2_-TG) (Cortell et al. 2010; Song et al. 2018). When 0.147 mg of free base of ergometrine is taken, a peak plasma concentration of 1.32 ng/ml (4 nM) was reached with a half-life of 1.4 h (De Groot et al.^1993^). Peroral ergometrine is completely absorbed with a bioavailability of 1.0 (De Groot et al. 1993). Taking these data and assuming a linear relationship between oral ergometrine and plasma ergometrine concentration, 1 µM ergometrine in plasma requires an absorption of about 37 mg of ergometrine that is only obtained in intoxications. However, ergometrine is a substrate of the drug-metabolizing enzyme called cytochrome CYP3A4 (Moubarak et al. 2003). Drugs that inhibit the activity of CYP3A4 could thus increase plasma levels of ergometrine. In other words, it is possible that under appropriate drug-drug interactions, even therapeutic dosages of ergometrine might lead to plasma concentrations of ergometrine that, based on the present data, might stimulate H_2_Rs in the human heart.

Ergometrine alone or in combination is sometimes used illicitly to induce hallucinations (Ott and Neely 1980). Hallucinations can occur with diagnostic injections of ergometrine used to perform stress echocardiographies (Selva et al. 1989). It is known that ergometrine can cause cardiac arrhythmias. These arrhythmias are usually explained by the constriction of coronary arteries by ergometrine via stimulation of serotonin receptors (review: Neumann et al. 2021a). Alternatively, based on the present study, ergometrine might stimulate H_2_Rs in human cardiomyocytes. Stimulation of H_2_Rs can lead to cardiac arrhythmias (review: Neumann et al. 2021a). We would predict that a tachycardia or other arrhythmias after treatment with ergometrine in patients could be blocked by cimetidine or famotidine, both of which are approved drugs. Nonetheless this prediction needs to be confirmed in a clinical study.

### Limitations of the study

We did not have the opportunity to study contractility and phosphorylation in human ventricle tissue for lack of access to that tissue. However, our data in Langendorff-perfused hearts provide first evidence that ergometrine could have ventricular effects, where H_2_Rs are also known to be present (Baumann et al. 1982). Hence, one would predict positive inotropic effects in the human ventricle. We cannot provide molecular information as to which parts of the ergometrine molecule can interact with the H_2_R. To this end, crystallographic studies would be required in subsequent work. We have no functional data whether in H2-TG the function of the β-adrenoceptor is elevated or decreased. That was beyond the scope of the present study.

## Conclusion

Ergometrine increases the force of contraction in cardiac preparations from H_2_-TG (not WT, nor 5-HT_4_-TG) and in human atrial preparations via H_2_ receptors.

## Data Availability

The data of this study are available from the corresponding author upon reasonable request.
